# Traumatic brain injury and the effects of diazepam, diltiazem, and MK-801 on GABA-A receptor subunit expression in rat hippocampus

**DOI:** 10.1186/1423-0127-17-38

**Published:** 2010-05-18

**Authors:** Cynthia J Gibson, Rebecca C Meyer, Robert J Hamm

**Affiliations:** 1 Department of Psychology, Washington College, Chestertown, MD, 21620, USA; 2 Neuroscience Program, Emory University, Atlanta, GA 30322, USA; 3 Department of Psychology, Virginia Commonwealth University, Richmond, VA 23284, USA

## Abstract

**Background:**

Excitatory amino acid release and subsequent biochemical cascades following traumatic brain injury (TBI) have been well documented, especially glutamate-related excitotoxicity. The effects of TBI on the essential functions of inhibitory GABA-A receptors, however, are poorly understood.

**Methods:**

We used Western blot procedures to test whether *in vivo *TBI in rat altered the protein expression of hippocampal GABA-A receptor subunits α1, α2, α3, α5, β3, and γ2 at 3 h, 6 h, 24 h, and 7 days post-injuy. We then used pre-injury injections of MK-801 to block calcium influx through the NMDA receptor, diltiazem to block L-type voltage-gated calcium influx, or diazepam to enhance chloride conductance, and re-examined the protein expressions of α1, α2, α3, and γ2, all of which were altered by TBI in the first study and all of which are important constituents in benzodiazepine-sensitive GABA-A receptors.

**Results:**

Western blot analysis revealed no injury-induced alterations in protein expression for GABA-A receptor α2 or α5 subunits at any time point post-injury. Significant time-dependent changes in α1, α3, β3, and γ2 protein expression. The pattern of alterations to GABA-A subunits was nearly identical after diltiazem and diazepam treatment, and MK-801 normalized expression of all subunits 24 hours post-TBI.

**Conclusions:**

These studies are the first to demonstrate that GABA-A receptor subunit expression is altered by TBI *in vivo*, and these alterations may be driven by calcium-mediated cascades in hippocampal neurons. Changes in GABA-A receptors in the hippocampus after TBI may have far-reaching consequences considering their essential importance in maintaining inhibitory balance and their extensive impact on neuronal function.

## Background

Traumatic brain injury (TBI) disrupts neuronal ionic balance and is known to produce glutamate-mediated neurotoxicity [1-3]. Glutamate related activation of N-methyl-D-aspartate (NMDA) receptors and the resulting elevations in intracellular calcium concentration ([Ca^2+^]_i_) are important components in synaptic and cellular degeneration and dysfunction after both *in vivo *[1,4,5] and *in vitro *neuronal injury [6-8]. Disruption of calcium (Ca^2+^) homeostasis after TBI has been implicated in a wide range of intracellular changes in gene expression, signaling pathways, enzymatic activation and even cellular death [see [9] for review]. Voltage gated calcium channels (VGCCs) also contribute to the increases in [Ca^2+^]_i _identified in glutamate related neurotoxicity due to TBI [10].

Although glutamate-related neurotoxic mechanisms after TBI have been studied extensively, relatively little is understood about inhibitory changes and the role of GABA receptors. Normal neuronal function relies on the constant orchestration and integration of excitatory and inhibitory potentials. GABA-A receptors (GABA_A_R) mediate the majority of inhibitory neurotransmission in the central nervous system by ligand gating of fast-acting chloride (Cl^-^) channels [11]. The impact of TBI on GABA_A_R is poorly understood even though changes in the composition and function of these receptors may have extensive consequences after injury.

The few available studies of GABA_A_R after TBI have resulted in an incomplete understanding of their contribution to injury-induced pathology, but have indicated that the receptor is affected by injury. Sihver et al. [12] found a decrease in GABA_A_R binding potential in the traumatized cortex and underlying hippocampus acutely (2 h) following lateral fluid percussion injury (FPI). Suppression of long term potentiation in the hippocampus has been demonstrated as early as 4 hours post-injury [13], although long term depression in the CA1 was not affected, and an overall hypoexcitation has been noted in early measures after TBI [14]. Contrary to the reduced inhibition in CA1 pyramidal cells [15] and CA3 to CA1 pathway [16] of the hippocampus, dentate gyrus granule cells [15] and the entorhinal cortex to dentate gyrus pathway demonstrated enhanced inhibition 2-15 days after fluid percussion TBI in rats [16]. Reeves et al. also noted that GABA immunoreactivity increased in the dentate gyrus and decreased in the CA1 two days after injury, correlating qualitatively with regional inhibitory changes. It is currently unknown whether changes in constituent GABA_A_R subtypes coincide with these functional changes in hippocampal inhibition.

GABA_A_R can be altered by changes in [Ca^2+^]_i_, indicating that the receptors are likely to be affected by glutamate-related excitotoxic effects of TBI. Specifically, Stelzer and Shi [17] found that NMDA and glutamate altered GABA_A_R currents in acutely isolated hippocampal cells, and this effect was dependent on the presence of Ca^2+^. Additionally, Matthews et al. [18] found the NMDA receptor antagonist MK-801 decreased GABA_A_R -mediated Cl^- ^uptake in the hippocampus. Lee et al. [10] found that the N-type VGCC blocker SNX-185 reduced the number of degenerating neurons when injected in the hippocampus following injury. Also, diltiazem, an FDA approved L-type VGCC antagonist, was discovered to be neuroprotective for cell culture retinal neurons when administered prior to injury [19]. Diltiazem and MK-801 were found to have synergistic effects, protecting against hypoxia-induced neural damage in rat hippocampal slices [20].

Also connecting [Ca^2+^]_i _and GABA_A_R function, Kao et al. [21] found that stretch injury of cultured cortical neurons resulted in increased Cl^- ^currents. These changes were blocked when an NMDA antagonist or a calcium/calmodulin protein kinase II (CaMKII) inhibitor were present in culture. CaMKII is known to be activated by increases in [Ca^2+^]_i _and is also known to phosphorylate GABA_A_R [22]. Kao et al. [21] suggested that injury-induced increases in glutamate activated NMDA receptors, increasing [Ca^2+^]_i _and subsequently activating CaMKII, resulting in altered GABA_A_R function due to phosphorylation of receptor proteins.

Although there is *in vitro *and indirect evidence that the GABA_A_R is altered by TBI, there are no *in vivo *studies identifying specific changes in GABA_A_R proteins. GABA_A_R typically form a pentameric structure consisting of five protein subunits surrounding a central Cl^- ^conducting ion pore. Although at least 16 subunits have been identified, along with several splice variants of the subunits, the most abundant subunits in the brain typically form a limited number of receptor combinations [23]. Reportedly, the following subunits combine to form nearly 80% of the GABA_A_R combinations in the rat brain: α1-3, β2-3, and γ2 [23-25], with α1β2γ2 and α2β2/3γ2 being the most abundant subunit combinations.

The current study utilized the *in vivo *FPI model to demonstrate that GABA_A_R subunit proteins are altered in the rat hippocampus after TBI. Expression of α1, α2, α3, α5, β3, and γ2 were measured by Western blot analysis 3 hours, 6 hours, 24 hours, and 7 days post-injury. These subunits are components in most of the GABA_A_R found in the hippocampus, and were chosen based on their relative abundance and their potentially important contributions in GABA_A_R function. When the expression of these proteins changed differentially due to TBI, the time point of greatest change for the greatest number of subunits (24 h) was chosen for pharmacological manipulation. The NMDA receptor antagonist MK-801, the L-type VGCC antagonist diltiazem, or the GABA_A_R agonist diazepam (DZ), was given prior to FPI to block Ca^2+ ^influx or enhance Cl- conductance. While MK-801 normalized all subunits measured 24 hours post-TBI, diltiazem and DZ were nearly identical in their impacts on the expression of GABA_A_R subunits.

## Methods

### Experimental Procedures

#### Subjects

Adult male Sprague-Dawley rats weighing approximately 320-340 g were used for all experiments (Harlan Laboratories; Indianapolis, IN). Animals were housed individually in a vivarium in shoebox-type cages on a 12:12 hour light/dark cycle. Animals in Study 1 were randomly assigned to either the sham or injured condition and to one of the following survival time points: 3 h, 6 h, 24 h, or 7 days (n = 3-4 per group, N = 32). Bilateral hippocampal tissue from each animal was used to analyze expression of all subunits. In Study 2, animals were randomly assigned to either sham or injured with a 24 hour survival time for each of the following treatments: no drug, MK-801 (Sigma-Aldrich), diltiazem (Henry Schein Veterinary), or DZ (Henry Schein) (n = 3-5 per group; N = 33). Animal care and experimental procedures were in accordance with the National Institute of Health Guide for the Care and Use of Laboratory Animals and the protocol was approved by the Institutional Animal Care and Use Committee at Creighton University, where the primary and secondary authors were both affiliated at the time of data collection.

#### Surgical Preparation and Injury

Animals were surgically prepared under sodium pentobarbital (48 mg/kg) 24 hours prior to injury, supplemented as needed with 1-3% isoflurane in a carrier gas of 70% N_2_O and 30% O_2 _to maintain the surgical plane. Animals were placed in a stereotaxic frame and a sagittal incision was made on the scalp. A craniotomy hole was drilled over the central suture, midway between bregma and lambda. Burr holes held two copper screws (56 × 6 mm) 1 mm rostral to bregma and 1 mm caudal to lambda. A modified Leur-Loc syringe hub (2.6 mm interior diameter) was placed over the exposed dura and sealed with cyanoacrylate adhesive. Dental acrylic was applied over the entire device to secure the hub to the skull (leaving the hub accessible). The incision was sutured and betadine and 1% lidocaine jelly (Henry Schein Animal Health) were applied to the wound. Animals were kept warm and continuously monitored until they fully recovered from the anesthesia.

A central (diffuse) injury was delivered twenty-four hours following the surgical preparation by a FPI device described in detail by Dixon et al., [26]. The FPI model in animals has been documented as the most common model of TBI [27], and the central injury was chosen as a diffuse option so bilateral hippocampi were equivalently injured. FPI in rats produces unconsciousness, cell damage to the vulnerable cortices and hippocampi, ionic cellular imbalance, excitotoxic cascades, blood flow changes, motor and memory deficits, and graded severity-dependent deficits consistent with human TBI [28,29]. Animals were anesthetized under 3.5% isoflurane in a carrier gas consisting of 70% N_2_O and 30% O_2_. The surgical incision was re-opened and the animals were connected to the fluid percussion device. Animals in the injury groups received a moderate fluid pulse (2.1 +/- 1 atm). Sham animals were attached to the injury device but no fluid pulse was delivered. The incision was sutured and betadine applied. Neurological assessments including tail, cornea, and righting reflexes were evaluated. The animals were closely monitored until they had sufficiently recovered and were then transferred back to the vivarium where food and water were available *ad libitum*.

### Western Blot Procedure

Animals were anesthetized under 3.5% isoflurane in a carrier gas of 70% N_2_O and 30% O_2 _at the time point indicated by the study design. The rats were quickly decapitated and bilateral hippocampi were dissected away on ice. The hippocampi were weighed and homogenized with a motorized homogenizer in a buffer consisting of 3 ml RIPA lysis buffer (US Biological; Swampscott, MA) and 30 μl Complete cocktail protease inhibitor (Roche Molecular Biochemicals; Mannheim, Germany) per gram of tissue.

The Western blot procedure was adapted from Kirkegaard & Perry Laboratories, Inc. (KPL; Gaithersburg, MD). Following homogenization, the hippocampi were centrifuged at 10,000 × g for 10 minutes. The supernatant was removed and spun a second time at 10,000 × g for 10 minutes. Aliquots of 10 μl of lysate (the supernatant) were stored at -20°C until used.

Following a BSA micro assay (Pierce, Rockford, IL) and spectrophotometry to assess protein levels, all treatment groups were run concurrently. Electrophoresis materials (e.g., gels, buffers, membranes) were Invitrogen's NuPage products (Carlsbad, CA), unless otherwise specified. All primary antibodies were polyclonal, purchased from Abcam Inc. (Cambridge, MA), and chemiluminescent reagents were purchased from KPL. Proteins were separated on pre-cast 4-12% Bis-Tris mini-gels using MOPS running buffer in the Novex Mini-Cell electrophoresis system. Separated proteins were then transferred to a nitrocellulose membrane (90 min at 30 V). Standard weights were run alongside each condition, including negative controls. Negative controls consisting of a lane that received all treatments, minus primary antibody, were included on all blots. Following transfer, the gel was stained with Coomassie FluorOrange (Invitrogen) to verify complete transfer to the membrane. Western blots were run using the KPL LumiGLO Reserve Chemiluminescence Kit. Primary antibody concentrations were empirically determined as follows: α1 = 1:500, α2 = 1:200, α3 = 1:150, β3 = 1:175, γ2 = 1:300. Several exposure times, ranging from 5 sec to 5 min were tested to determine the clearest visualization. Digital images were scanned and saved from the developed films. Following immunoblotting, membranes were stained with SYPRO Ruby stain (Sigma Aldrich, St. Louis MO) to ensure even loading of proteins across lanes.

No protein bands were visible on any blots run under minus primary conditions. Gel staining following protein transfer indicated that proteins were transferred equivalently across lanes. Blots revealing uneven distribution of protein were excluded from the studies.

### Drug Administration

All drugs were administered 15 minutes prior to TBI. NMDA-mediated Ca^2+ ^influx was blocked by administration of 0.3 mg/kg MK-801 (Tocris; Ellisville, MO) in saline solution. This dose was previously shown to be protective against motor deficits [2] and cognitive deficits following fluid percussion TBI alone [30] or in combination with secondary bilateral entorhinal cortex lesions [31]. Ca^2+ ^influx through L-type VGCCs was blocked with 5 mg/kg diltiazem, an FDA-approved drug specific to L-type channels. Chloride conduction through the GABA_A_R was enhanced using 5 mg/kg DZ, a pretreatment dose previously shown to be neuroprotective against cognitive deficits after TBI [32].

### Statistical Analysis

Protein bands of approximately 60 kDa (α1), 53 kDa (α2), 53 kDa (α3), 51 kDa (α5), 50 kDa (β3), and 45 kDa (γ2) were identified and quantified for optical density using IMT i-Solution, Inc. software (Image and Microscope Technology). Due to gel size constraints not all subjects in a group could be run on the same blot, so data were normalized as follows. At least 2 or more sham, untreated lanes were included on all blots. Relative optical density (ROD) of each individual protein band was quantified as a percent difference from the value of the mean sham density for each blot, where the mean sham density was normalized at 100. Therefore, OD measurements for each band in both studies were defined in ROD units, relative to the mean sham OD per blot.

Study 1 results from α1, α3, β3, and γ2 subunits were analyzed separately using a 2 (TBI or sham) × 4 (time) factorial ANOVA. For α2 and α5 subunits, the 6 hour time point was excluded based on lack of changes in all other time points, so separate 2 (TBI or sham) × 3 (time) factorial ANOVAs were used for analysis. In order to determine which time point produced the greatest change, a Fisher's LSD post-hoc was used for time point comparisons for each subunit. The results of this analysis indicated that the 24 hour post-injury time point revealed the greatest changes across the most subunits. Therefore, in Study 2 the effects of pre-injury treatment with MK-801, diltiazem, or DZ on protein expression 24 hours following injury were determined using a one-factor ANOVA and Fisher's LSD post-hoc to compare group differences (sham-untreated, sham-treated, injured-untreated, and injured treated) for each of the 3 drug treatments. Due to the relative importance of γ2 and the various α subunits to BZ-type GABA_A_R pharmacological function, α1, α2, α3, and γ2 were chosen for inclusion in Study 2. All drug treatment groups were run concurrently with untreated sham and injured groups during Western blot procedures to control for variation in group effects.

## Results

### Neurological Recovery from TBI

Analyses by ANOVA revealed that recovery of reflexes (corneal blink, tail pinch, righting reflex), measured in minutes, was significantly suppressed in the injured groups compared to the sham groups. All experimental groups demonstrated equivalent injuries as measured by atm and reflex suppression (data not shown).

### Study 1: Expression of GABA_A_R Subunits After TBI

**Figure 1:**
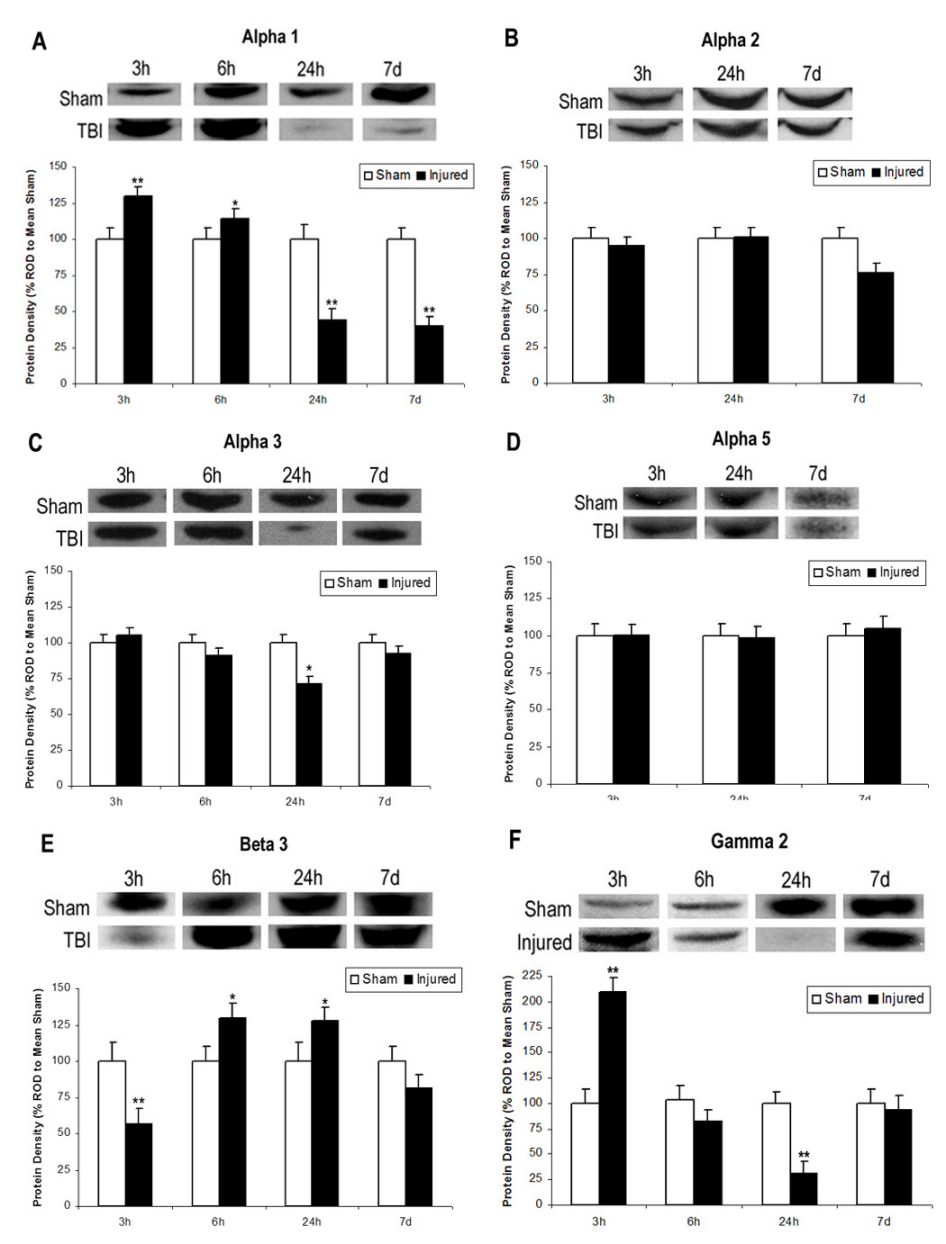
**Expression of GABA_A_R Subunits After TBI**. Western blot analysis of GABA-A receptor subunits α1, α2, α3, α5, β3, and γ2 in the hippocampus 3 h, 6 h, 24 h, or 7 days after TBI. Histograms of protein expression were measured in Relative Optical Density (ROD) proportions normalized against the mean sham OD for each individual blot. Asterisks indicate significant differences based on factorial ANOVA; *p < .05, **p < .01. Error bars represent +/-SEM. **A: **Alpha 1 demonstrated significantly increased expression 3 h and 6 h post-TBI followed by significantly decreased expression at 24 h and 7 days. **B: **There were no significant differences in Alpha 2. **C: **Alpha 3 demonstrated significantly decreased expression at 24 h post-TBI only. **D: **There were no significant differences in Alpha 5. **E: **Beta 3 demonstrated initially significant decreased expression at 3 h, followed by significantly increased expression at 6 h and 24 h post injury. **F: **Gamma 2 demonstrated significantly increased expression at 3 h and significantly decreased expression at 24 h post-TBI.

No significant differences were found between sham and injured animals for α2 or α5 relative protein densities at any time point (Figure 1). Expression of α1 ROD in injured hippocampus was significantly higher at 3 hours (*M *= 129.72) and 6 hours (*M *= 114.34 and significantly lower at 24 hours (*M *= 44.23) and 7 days (*M *= 39.81) compared to sham (*M *= 100) [*F*(3,18) = 18.329, *p *< .001].

Expression of α3 subunit ROD in injured hippocampus was significantly reduced at 24 hours (*M *= 74.47) compared to sham [*F*(3,20) = 3.62, *p *< .05)]. No other time points for α3 were significantly different between injured and sham.

Expression of β3 subunit ROD in injured hippocampus was significantly lower at 3 hours (*M *= 74.97) and significantly higher at 6 hours (*M *= 114.87) and 24 hours (*M *= 118.46) compared to sham [*F*(3,16) = 5.319, *p *= .01]. There was no difference between injured and sham measures at 7 days post-injury.

Expression of γ2 subunit ROD for injured hippocampus was significantly higher at 3 hours (*M *= 155.03) and significantly lower at 24 hours (*M *= 69.09) compared to sham [*F*(3,21) = 15.827, *p *< .001). There were no differences in γ2 expression between injured and sham at 6 hours or 7 days post-injury.

### Study 2: Relative Optical Density of GABA_A_R Subunits after pre-TBI Drug Treatment

**Figure 2:**
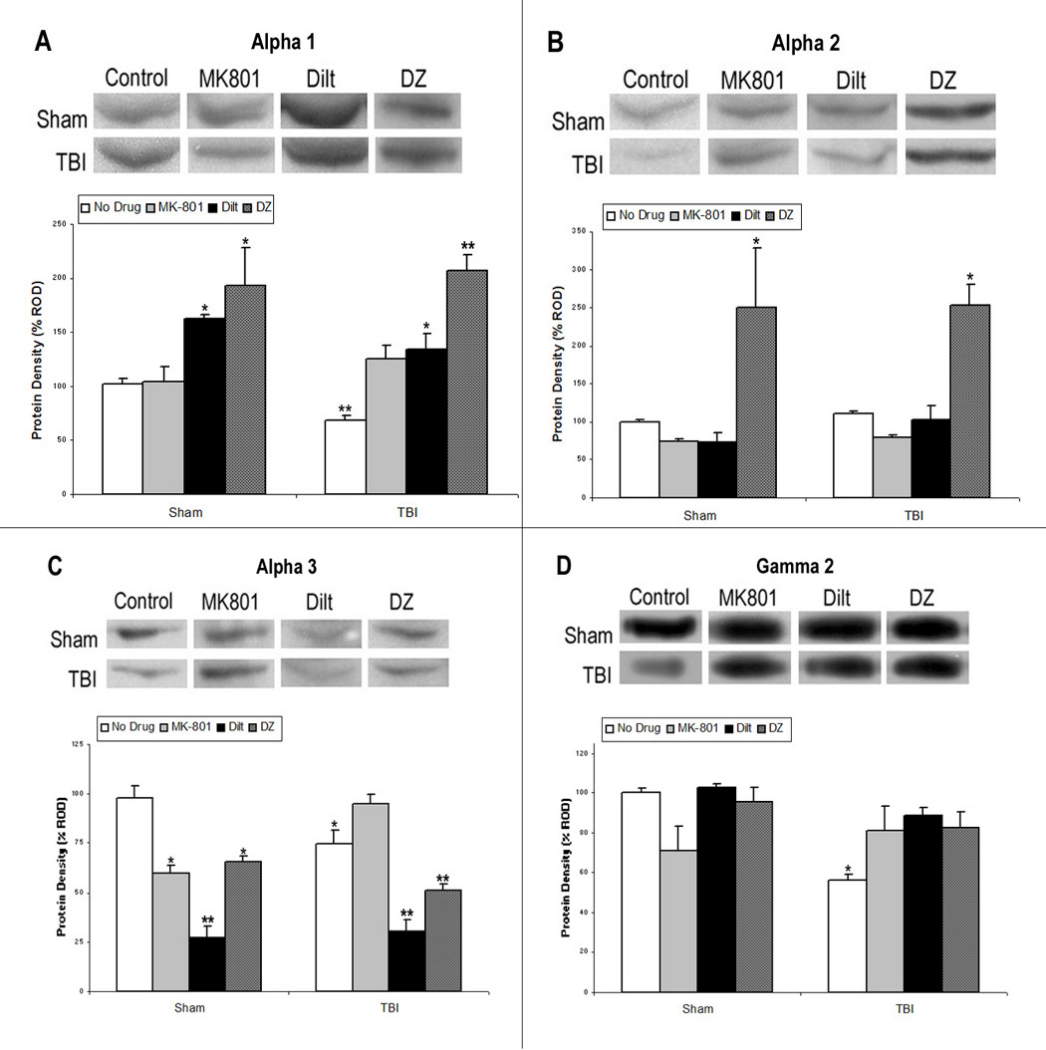
**GABA_A_R Subunits After pre-TBI Drug Administration**. Western blot analysis of GABA-A receptor subunits α1, α2, α3, and γ2 in the hippocampus 24 h post-TBI with either no drug (untreated), MK-801 (NMDA calcium blocker, 0.3 mg/kg), diltiazem (L-type VGCC antagonist, 5 mg/kg), or diazepam (GABA-A agonist, 5 mg/kg). Histograms of protein expression were measured in Relative Optical Density (ROD) proportions normalized against the mean sham OD for each individual blot. Each blot contained at least 2 sham and 1 injured untreated protein lanes. Tissue from the same sham and injured groups was used for comparison to each drug by ANOVA. Asterisks indicate significant differences based on factorial ANOVA; *p < .05, **p < .01. Error bars represent +/-SEM. **A: **MK-801 normalized alpha 1 expression, while diltiazem and diazepam significantly increased expression in both sham and injured animals. **B: **No alpha 2 injury effects or drug effects were found for MK-801 or diltiazem, although diazepam significantly increased alpha 2 expression in both sham and injured animals. **C: **MK-801 significantly decreased alpha 3 expression in sham but not injured animals, while diltiazem and diazepam significantly decreased expression in both sham and injured animals. **D: **Gamma 2 expression was normalized by all drug treatments.

MK-801 pre-injury administration prevented the significant reduction in α1, α3, and γ2 ROD 24 hours post-injury. MK-801 had no significant effect on measures of sham protein expression for α1 or γ2, although it significantly decreased sham α3 expression [*F*(3,9) = 7.484, *p *< .01]. MK-801 had no significant effect on α2 expression. Table 1 summarizes significant group changes in protein expression, while Figure 2 presents representative blots and significant changes for each subunit.

**Table 1:** Summary of significant changes in GABAAR subunit ROD 24 hours after TBI or Sham injury

	α1	α2	α3	γ2
Sham-Untreated	-	-	-	-
Injured-Untreated	↓	-	↓	↓
Sham+MK801	-	-	↓	-
Injured+MK801	-	-	-	-
Sham+diltiazem	↑	-	↓↓	-
Injured+diltiazem	↑	-	↓↓	-
Sham+DZ	↑	↑	↓↓	-
Injured+DZ	↑	↑	↓↓	-

Double arrows indicate a drug-induced significant change beyond effects due to TBI only (i.e, compared to the injured untreated group). DZ and Diltiazem treatment had identical patterns of significance for all subunits except α2, which had significantly increased expression due to DZ treatment. MK-801 normalized all TBI-induced significant changes in protein expression.

Diltiazem not only prevented the significant decrease in α1 ROD at 24 hours post-injury, but significantly increased α1 expression in both injured (*M *= 162.67) and sham (*M *= 133.90) compared to untreated sham [*F*(3,8) = 11.364, *p *< .01], indicating diltiazem significantly increased α1 expression, regardless of injury condition. Diltiazem significantly decreased α3 ROD in both injured (*M *= 30.48) and sham (*M *= 27.38), beyond the significant decrease seen in untreated injured hippocampus (*M *= 74.93) [*F*(3,9) = 34.13, p < .001], indicating diltiazem significantly decreased α3 expression, regardless of injury condition. Diltiazem normalized the significant decrease in γ2 expression due to injury, but had no effect on γ2 in sham hippocampus. Diltiazem had no significant effect on α2 expression.

The effects of DZ on α1, α3, and γ2 expression were the same as the effects of diltiazem on these subunits. DZ significantly increased both sham (*M *= 193.48) and injured (*M *= 207.19) α1 ROD 24 hours post-injury [*F*(3,8) = 19.624, *p *< .001], indicating DZ significantly increased α1 expression, regardless of injury condition. DZ also significantly reduced α3 ROD in both sham (*M *= 65.65) and injured (*M *= 50.93) hippocampus beyond the injury-induced decrease in expression (*M *= 74.93) [*F*(3,9) = 14.907, *p *< .01], indicating DZ decreased α3 expression, regardless of injury condition. DZ normalized γ2 injury-induced decreases in ROD without significantly effecting sham γ2 expression. DZ had the unique effect of significantly increasing α2 expression in both sham (*M *= 249.62) and injured (*M *= 252.89) hippocampal tissue, indicating DZ significantly increased α2 expression, even though there was no injury effect on this subunit.

## Discussion

The hypothesis that TBI would differentially alter GABA_A_R subunit expression in the hippocampus in a time-dependent manner was supported. Both α1 and γ2 subunit expression increased acutely after injury, but were significantly decreased by 24 h, while α3 and β3 showed time-specific transient changes and α2 and α5 subunits were not altered significantly at any time point. MK-801 prevented changes to all subunits studied 24 hours after TBI, while diltiazem and DZ treatments had nearly identical effects, normalizing γ2 and altering α1 and α3 expression. DZ also significantly increased α2 expression in both sham and injured animals.

### Study 1: Expression of GABA_A_R Subunits After TBI

This study is the first to demonstrate time-dependent *in vivo *GABA_A_R protein expression changes due to TBI. Most predominant GABA-A subunits have been identified as having specific physiological relevance, often through the use of knockout and knockdown animals. Differential changes in subunits may have important relevance since GABA_A_R subunits regulate different functions. The β subunit of the GABA_A_R is vital for the regulation of ion selectivity and general properties of the chloride channel [33,34], as evidenced by β3 knockout mice developing epilepsy, a disorder associated with a disruption in the ionic balance in the cells [25]. The β subunits also differentially regulate inhibitory Cl^- ^flow [35]. The transitory increase in β3 expression at 3 hours and decrease at 6 and 24 hours post-injury may be related to time-dependent alterations in inhibitory functioning, although further measures of other β subunits and their influence on inhibitory function are still needed.

The γ subunit differentially regulates benzodiazepine (BZ) sensitivity with γ2 knockdown mice showing reduced BZ binding [36] and γ1 and γ3 not demonstrating any BZ activity [37]. The γ2 subunit is also endogenously required for the clustering of receptors at the synapse [38]. Therefore, the initial increase in γ2 expression 3 hours post-TBI, followed by a decrease at 24 hours may indicate greater γ2-containing GABA_A_R clustering and greater BZ binding potential during the first few hours after injury, therefore providing a widow of initial therapeutic sensitivity for BZ treatment post-TBI.

The α subunit of the GABA_A_R is important for post-synaptic signaling of the receptors [39] and specific effects of BZs such as DZ [40]. Additionally, the various α subunits have a wide range of unique functions. Constrained mainly to hippocampal neurons, α5 regulates hippocampal dendritic pyramidal inhibition related to learning and memory plasticity [41]. Since hippocampally-driven deficits in learning and memory are well demonstrated after TBI, it is important to note that α5 subunit expression did not change at any time point studied. This may indicate relative stability of this subunit, or the changes may be regionally specific and therefore not detected in the whole hippocampal homogenate used in this study. The α3 subunit contributes to GABAergic inhibition of dopamine neurons, and genetic ablation of α3 subunits is found to cause disruptions in sensory gating as measured through pre-pulse inhibition of acoustic startle [41]. Since decreases in α3 were found only at the 24 hour time point, this may indicate a time-dependent fluctuation in GABA-dopamine interaction during the shift from acute to chronic post-injury measurements. Found mainly in the amygdala, α2 exerts some control over emotional functioning [42], which may help explain its anxiolytic role in BZ action [25]. Additionally, the α2 subunit is highly expressed in the ventral hippocampus which has been found to exert weaker inhibitory tone and has higher seizure susceptibility compared to the dorsal hippocampus, which primarily expresses α1 [35]. This study focused on mild/moderate TBI and no post-injury seizure activity was detected, which may partially explain why α2 expression was unaffected. However, deficits in excitatory/inhibitory balances in neurotransmission in the hippocampus have been found even in mild TBI, and were believed to contribute to both increased seizure susceptibility and cognitive deficits [43].

The most widespread α subunit with the most diversely documented functional implications is α1, which is highly expressed in the dorsal hippocampus where it likely contributes to greater GABA binding and lower seizure susceptibility [35]. Also, α1 plays an important role in development [44], so changes in α1 subunit levels may be indicative of a partial reversion of certain GABAergic receptors to a more developmental state. This is an intriguing possibility since GABA activity can be excitatory during development [45]. Excitatory GABAergic signaling has already been proposed as a contributor to the pathophysiology of epilepsy [46]. Persistent alterations in inhibitory balance after TBI have been implicated in increased post-injury development of epilepsy [43,47,48] and in cognitive memory deficits [43,49,15].

Just as different subunits have unique effects on GABA-A function, their differential alteration following TBI can have specific implications for the pathophysiological state of the recovering brain. Thompson et al. [50] demonstrated differential mRNA changes for GABA_A_R subunits in cultured cerebellar granule cells exposed to protein kinase A. Protein kinase A inhibitors prevented these effects on α1 but not on α6, indicating differential regulatory mechanisms for different subunits. Epilepsy research has also demonstrated disparate alterations in subunits. Although β3 mRNA decreased in the hippocampus following kainic acid-induced seizures, α1 mRNA increased in the interneurons of the dentate gyrus and CA3 [51]. Huopaniemi et al. [52] demonstrated more than 130 transcriptional changes in α2, α3, and α5 in α1 point-mutated mice after a single DZ injection, although there was no effect in wild type mice. Therefore, in the absence of specific α1 genes, other α subunit transcripts changed, indicating a complicated compensatory relationship among α subunits [52].

The α1 subunit was the only one to demonstrate significant changes at every time point studied. This is important because α1 may mediate apoptosis via the endoplasmic reticulum (ER) stress pathway [53]. Since the cells in the current study were lysed to obtain whole protein measures, regional specificity of each protein cannot be determined and therefore the changes may also represent subunits in the ER. Overexpression of α1 may be related to apoptotic processes after TBI. Also, α1 overexpression can trigger apoptosis due to a complicated relationship with c-myc, a proto-oncogene that regulates cellular proliferation and apoptosis. The α1 gene is a direct target for suppression by c-myc and mRNA expression is inversely related to c-myc expression. This inverse balance between c-myc and α1 may be either a marker or a key player in the developmental cessation of neuronal pruning. Shifts in c-myc expression during neuronal insult such as TBI may result in changes to the α1 gene, independent of its role in GABA_A_R function. Over-expression of α1 has also been associated with apoptosis in a Ca^2+^-dependent manner. Specifically, disruption of ER Ca^2+ ^balance may alter α1 mRNA, producing an increase that activates caspase 3 and induces apoptosis [53]. Therefore, blocking Ca^2+ ^influx due to TBI may prevent α1-associated apoptosis by preventing significant increases in α1 subunit proteins.

Alterations in α1 expression may also affect the function of the GABA_A_R. Increases in α1 mRNA and protein expression during development correspond to increases in BZ binding and altered zinc sensitivity [54], while reduced α1 mRNA and protein expression in the hippocampus of seizure-prone animals is associated with reduced inhibitory tone [55]. Increased α1 subunits may also contribute to pronounced sedative or amnesic effects associated with BZs without any effect on the anxiolytic or relaxant properties [56,57]. Since changes in α1 protein expression were different during acute (3 h, 6 h) and chronic (24 h, 7 day) post-injury time points, there may be important implications for the timing of BZ use in TBI patients. O'Dell and Hamm [58] found that DZ administered around the time of injury significantly improved mortality and cognitive outcome in a water maze task 2 weeks after FPI. However, chronic treatment with Suritozole, a negative GABA_A_R modulator similar to BZ inverse agonists, starting 24 hours post injury was also cognitively beneficial in the water maze [58]. Increased α1 and γ2 within 3 hours of TBI in this study may indicate a shift in GABA_A_R constituent proteins to a more BZ-sensitive composition. This could prime GABA_A_R for greater sensitivity to BZs and increased Cl^- ^conductance, while the decrease in α1 and γ2 at 24 hours may be an attempt to compensate for chronic hypofunctioning by reducing GABA_A_R sensitivity. Novel compounds that target specific subtypes or subunits of GABA_A_R may provide more insight into their roles after TBI.

Alteration to GABA_A_R expression may be due to phosphorylation of subunit proteins. Increases in glutamate after injury trigger VGCC and NMDA receptors, increasing [Ca^2+^]_i_, and resulting in activation of CaMKII. A single dose of DZ can downregulate CaMKIIα transcription quickly and persistently, although this downregulation of CaMKIIα transcripts in wild type mice is not found in mice with point mutations to GABA_A_R α1 [52]. Folkerts et al. [59] found that fluid percussion TBI increased both CaMKIIα and total CaMKII in the hippocampus, although this increase was transient with CaMKII elevations no longer significant by 3 hours post-injury. Protein phosphatases such as calcineurin also increase after TBI due to activation by elevated [Ca^2+^]_i _[60]. Folkerts et al. [59] proposed protein phosphatase activation, including calcineurin, may explain the unusual pattern of CaMKII immunostaining in CA3 pyramidal cells in the hippocampus after TBI.

### Study 2: GABA_A_R Subunits After pre-TBI Drug Administration

A single pre-injury injection of MK-801 normalized GABA_A_R subunit expression. Therefore, blockade of Ca^2+ ^influx through the NMDA receptor effectively attenuated α1, α3, and γ2 subunit decreases 24 hours post-injury, indicating that injury-related influx of Ca^2+ ^through the NMDA receptor contributed to the changes in GABA-A subunit expression. There may be diverse regulatory mechanisms involved in the interaction between NMDA receptors and GABA_A_R. Kim et al. [61] found chronic blockade of Ca^2+ ^influx through the NMDA receptor with MK-801 reduced β3 and increased β2 mRNA but not protein expression in the hippocampus. Chronic MK-801 treatment did not alter GABA-A α1 or NMDA receptor subunit mRNA or protein expression in the hippocampus but GABA_A_R-mediated Cl^- ^uptake was still significantly decreased [18].

Although this study was not designed to determine the specific mechanism by which elevated [Ca^2+^]_i _resulted in alterations to GABA_A_R subunits, we do know that elevated [Ca^2+^]_i _alters numerous intracellular mechanisms following TBI [9], including activation of apoptotic factors, CaMKII, and protein phosphatases. Although Ca^2+ ^influx through the NMDA receptor is a major source of neuronal excitotoxicity [6], other sources of Ca^2+ ^influx may also be important. For example, VGCC blockers have been shown to be beneficial after TBI [10,62].

Diltiazem, an L-type VGCC blocker, and DZ, a GABA_A_R agonist, had statistically identical effects on the expression of GABA_A_R subunits α1, α3, and γ2, normalizing γ2 and significantly increasing α1 and decreasing α3. Changes to α1 and α3 occurred in both sham and injured animals, indicating drug effects that overrode the injury effects. Some L-type channel blockers have known effects on receptors such as NMDA [63] or GABA-A [64], but diltiazem has been shown to have no direct effect on recombinant α1β2γ2 receptors [65]. However, VGCC regulation of GABA_A_R surface expression may be a common mechanism since it has been implicated after hypoxia [66] and extended GABA exposure [67]. Therefore, the similar profiles of GABA_A_R changes for diltiazem and DZ are likely due to similarities of action that alter excitatory/inhibitory balance, rather than a direct effect on the GABA_A_R.

Both diltiazem and DZ inhibit Ca^2+ ^release induced by sodium presence in rat brain mitochondria [68] by inhibiting mitochondrial Ca^2+ ^efflux via the sodium/calcium exchanger [68,69]. One method of buffering excessive increases in [Ca^2+^]_i _after TBI is to sequester Ca^2+ ^into organelles such as the mitochondria. Calcium, however, can damage the mitochondria, resulting in several detrimental consequences, including the release of pro-apoptotic factors [9]. Through the enhancement of GABA-A Cl^- ^influx, DZ regulates Ca^2+ ^and apoptotic factor release from the mitochondria, providing neuroprotection after *in vivo *ischemia and *in vitro *glutamate or oxidative stress in CA1 hippocampal and brain slices, respectively [70]. This DZ regulation of mitochondrial Ca^2+ ^release likely plays an important role *in vivo *after TBI as well.

Diltiazem and MK-801 have synergistic neuroprotection against hypoxia in rat hippocampal slices, beyond simple additive effects [20]. Diltiazem [71] and MK-801 [72] both reduced excitotoxic effects of glutamate and NMDA exposure in a cell culture model of hypoxia. Although diltiazem did not block NMDA receptors, it was more effective in reducing NMDA-mediated than glutamate-mediated Ca^2+ ^influx, and was more effective at lower doses than MK-801 at regulating glutamate-mediated Ca^2+ ^influx. The effectiveness of diltiazem highlights the importance of non-NMDA sources of intracellular Ca^2+ ^influx. Opening of VGCCs can trigger removal of the NMDA receptor magnesium blockade, with NMDA receptor-mediated influx of Ca^2+ ^further depolarizing VGCCs. Diltiazem, therefore, blocks L-type VGCCs at initial and continuing stages of Ca^2+ ^entry. Due to relative safety and potential benefits, both diltiazem and DZ may have therapeutic potential acutely following TBI, but more information is needed to understand the mechanism of neuroprotection, influence on cascades, and impact on behavioral outcome. Evidence indicates the timing of administration of these drugs will be crucial.

## Conclusions

The current studies are the first to demonstrate that TBI induces time-dependent changes in GABA_A_R α1, α3, β3, and γ2, but not α2 or α5 expression during the first 7 days after injury. The changes in GABA_A_R protein expression found in these studies may have important consequences for post-injury apoptosis in the hippocampus, as well as neuronal excitability and pharmacological responsiveness after TBI. These studies, therefore, support the hypotheses that TBI alters the constituent proteins of the GABA_A_R and that these alterations may be driven by a calcium-mediated mechanism.

## Competing interests

The authors declare that they have no competing interests.

## Authors' contributions

CG conceived of the design of the studies, served as PI for grant funding, conducted all surgery and injury procedures, contributed to Western blot procedures, performed all data and statistical analyses, and was primary contributor to the final manuscript. RM contributed to refinement of the design, assisted with all surgical and injury procedures, performed Western blot procedures, and helped draft the manuscript. RH contributed to the initial conception and design. All authors contributed to and approved the final manuscript.

## References

[B1] Faden AL Demediuk P Panter SS Vink R The role of excitatory amino acids and NMDA receptors in traumatic brain injury Science 1989 244 798 800 10.1126/science.2567056 2567056

[B2] Hayes RL Jenkins LW Lyeth BG Neurotransmitter-mediated mechanisms of TBI: Acetylcholine and excitatory amino acids J Neurotrauma 1992 9 S173 S187 10.1089/neu.1992.9.173 1350312

[B3] Katayama Y Becker DP Tamura T Hovda DA Massive increases in extracellular potassium and the indiscriminate release of glutamate following concussive brain injury J Neurosurg 1990 73 889 900 10.3171/jns.1990.73.6.0889 1977896

[B4] Goda M Isono M Fujiki M Kobayashi H Both MK801 and NBQX reduce the neuronal damage after impact-acceleration brain injury J Neurotrauma 2002 19 1445 1456 10.1089/089771502320914679 12490009

[B5] Hayes RL Jenkins LW Lyeth BG Balster RL Robinson SE Clifton GL Stubbins JF Young AF Pretreatment with phencyclidine, an N-methyl-D-aspartate antagonist, attenuated long-term behavior deficits in rat produced by traumatic brain injury J Neurotrauma 1998 5 259 274 10.1089/neu.1988.5.259 2854855

[B6] Bading H Segal MM Sucher NJ Dudek H Lipton SA Greenberg ME N-methyl-D-aspartate receptors are critical for mediating the effects of glutamate on intracellular calcium concentration and immediate early gene expression in cultured hippocampal neurons Neuroscience 1995 64 653 664 10.1016/0306-4522(94)00462-E 7715778

[B7] Choi DW Ionic dependence of glutamate neurotoxicity J Neursoci 1987 7 369 379 10.1523/JNEUROSCI.07-02-00369.1987 PMC6568907 2880938

[B8] Weber JT Razigalinski BA Willoughby KA Moore SF Ellis EF Alterations in calcium-mediated signal transduction after traumatic injury of cortical neurons Cell Calcium 1999 26 289 299 10.1054/ceca.1999.0082 10668567

[B9] Weber JT Calcium homeostasis following traumatic neuronal injury Curr Neurovasc Res 2004 1 151 171 10.2174/1567202043480134 16185191

[B10] Lee LL Galo E Lyeth BG Muizelaar P Berman RF Neuroprotection in the rat lateral fluid percussion model of traumatic brain injury by SNX-185, an N-type voltage-gated calcium channel blocker Exp Neurol 2004 190 70 78 10.1016/j.expneurol.2004.07.003 15473981

[B11] Mohler H Fritschy JM Luscher B Rudolph U Benson J Benke D Narahashi T The GABA-A receptors: from subunits to diverse functions Ion Channels 1996 4 Plenum Press 8744207

[B12] Sihver S Marklund N Hillered L Langstrom B Watanabe Y Bergstrom M Changes in mACh, NMDA and GABA-A receptor binding after lateral fluid-percussion injury: *in vitro *autoradiography of rat brain frozen sections J Neurochem 2001 78 417 423 10.1046/j.1471-4159.2001.00428.x 11483644

[B13] Sick TJ Perez-Pinzon MA Feng ZZ Impaired expression of long-term potentiation in hippocampal slices 4 and 48 hours following mild fluid-percussion brain injury in vivo Brain Res 1998 785 287 292 10.1016/S0006-8993(97)01418-2 9518654

[B14] D'Ambrosio R Maris DO Grady MS Winn HR Janigro D Selective loss of hippocampal long-term potentiation, but not depression, following fluid percussion injury Brain Res 1998 786 64 79 10.1016/S0006-8993(97)01412-1 9554957

[B15] Witgen BM Lifshitz J Smith ML Schwarzbach E Liang SL Grady MS Cohen AS Regional hippocampal alteration associated with cognitive deficit following experimental brain injury: a systems, network and cellular evaluation Neuroscience 2005 133 1 15 10.1016/j.neuroscience.2005.01.052 15893627

[B16] Reeves TM Lyeth BG Phillips LL Hamm RJ Povlishock JT The effects of traumatic brain injury on inhibition in the hippocampus and dentate gyrus Brain Res 1997 757 119 132 10.1016/S0006-8993(97)00170-4 9200506

[B17] Stelzer A Shi H Impairment of GABAA receptor function by N-methyl-D-aspartate-mediated calcium influx in isolated CA1 pyramidal cells Neuroscience 1994 62 813 828 10.1016/0306-4522(94)90479-0 7870309

[B18] Matthews DB Dralic JE Devaud LL Fritschy JM Marrow AL Chronic blockade of N-methyl-D-aspartate receptors alters gamma-aminobutyric acid A receptor peptide expression and function in the rat J Neurochem 2000 74 1522 1528 10.1046/j.1471-4159.2000.0741522.x 10737609

[B19] Vallazza-Deschamps G Fuchs C Cia D Tessier LH Sahel JA Dreyfus H Picaud S Diltiazem-induced neuroprotection in glutamate excitotoxicity and ischemic insult of retinal neurons Doc Opthalmal 2005 110 25 35 10.1007/s10633-005-7341-1 16249955

[B20] Schurr A Payne RS Rigor BM Synergism between diltiazem and MK-801 but not APV in protecting hippocampal slices against hypoxic damage Brain Res 1995 684 233 236 10.1016/0006-8993(95)00466-4 7583230

[B21] Kao C-Q Goforth PB Ellis EF Satin LS Potentiation of GABAA currents after mechanical injury of cortical neurons J Neurotrauma 2004 21 259 270 10.1089/089771504322972059 15115601

[B22] Swope SL Moss SI Raymond LA Huganir RL Regulation of ligand-gated ion channels by protein phosphorylation Adv Second Messenger Phosphoprotein Res 1999 33 49 78 10218114 10.1016/s1040-7952(99)80005-6

[B23] Whiting PJ GABA-A receptor subtypes in the brain: a paradigm for CNS drug discovery? Drug Discov Today 2003 8 445 450 10.1016/S1359-6446(03)02703-X 12801796

[B24] McKernan RM Whiting PJ Which GABAA-receptor subtypes really occur in the brain? Trends Neurosci 1996 19 139 143 10.1016/S0166-2236(96)80023-3 8658597

[B25] Sieghart W Sperk G Subunit distribution, composition and function of GABA-A receptor subtypes Curr Top Med Chem 2002 2 795 816 10.2174/1568026023393507 12171572

[B26] Dixon CE Lyeth BG Povlishock JT Findling RL Hamm RJ Marmarou A Young HF Hayes R A fluid percussion model of experimental brain injury in the rat J Neurosurg 1987 67 110 119 10.3171/jns.1987.67.1.0110 3598659

[B27] Thompson HJ Lifshitz J Marklund N Grady MS Graham DI Hovda DA McIntosh TK Lateral fluid percussion brain injury: A 15-year review and evaluation J Neurotrauma 2005 22 42 75 10.1089/neu.2005.22.42 15665602

[B28] Gennarelli TA Animate models of human head injury J Neurotrauma 1994 11 357 368 10.1089/neu.1994.11.357 7837277

[B29] Povlishock JT Hayes RL Michel ME McIntosh TK Workshop of animal models of traumatic brain injury J Neurotrauma 1994 11 723 732 10.1089/neu.1994.11.723 7723071

[B30] Hamm RJ O'Dell DM Pike BR Lyeth BG Cognitive impairment following traumatic brain injury: the effects of pre- and post-injury administration of scopolomine and MK-801 Brain Res Cogn Brain Res 1993 1 223 226 10.1016/0926-6410(93)90006-Q 8003921

[B31] Phillips LL Lyeth BG Hamm RJ Reeves TM Povlishock JT Glutamate antagonism during secondary deafferentation enhances cognition and axo-dendritic integrity after traumatic brain injury Hippocampus 1998 8 390 401 10.1002/(SICI)1098-1063(1998)8:4<390::AID-HIPO7>3.0.CO;2-L 9744424

[B32] O'Dell DM Gibson CJ Wilson MS DeFord MD Hamm RJ Positive and negative modulation of the GABA_A _receptor and outcome after traumatic brain injury in rats Brain Res 2000 861 325 332 10.1016/S0006-8993(00)02055-2 10760494

[B33] Jensen ML Timmermann DB Johansen TH Schousboe A Varming T Ahring PK The beta subunit determines the ion selectivity of the GABAA receptor J Biol Chem 2002 277 41438 41447 10.1074/jbc.M205645200 12177063

[B34] Ymer S Schofield PR Draguhn A Werner P Kohler M Seeburg PH GABAA receptor beta subunit heterogeneity: functional expression of cloned cDNAs EMBO Rep 1989 8 1665 1670 10.1002/j.1460-2075.1989.tb03557.x PMC401007 2548852

[B35] Sotiriou E Papatheodoropoulos C Angelatou F Differential expression of gamma-aminobutyric-acid-A receptor subunits in rat dorsal and ventral hippocampus J Neurosci Res 2005 82 690 700 10.1002/jnr.20670 16273537

[B36] Chandra D Korpi ER Miralles CP DeBlas AL Homanics GE GABA_A _receptor γ2 subunit knockdown mice have enhanced anxiety-like behavior but unaltered hypnotic response to benzodiazepines BMC Neuroscience 2005 6 30 10.1186/1471-2202-6-30 15850489 PMC1097738

[B37] Olsen RW Sieghart W GABAA receptors: Subtype provide diversity of function and pharmacology Neuropharmacol 2009 56 141 148 10.1016/j.neuropharm.2008.07.045 PMC3525320 18760291

[B38] Essrich C Lorez M Benson JA Fritschy JM Luscher B Postsynaptic clustering of major GABAA receptor subtypes requires the gamma 2 subunit and gephyrin Nat Neurosci 1998 1 563 571 10.1038/2798 10196563

[B39] Lavoie AM Tingey JJ Harrison NL Pritchett DB Twyman RE Activation and deactivation rates of recombinant GABAA receptor channels are dependent on alpha-subunit isoform Biophysical Journal 1997 73 2518 2526 10.1016/S0006-3495(97)78280-8 9370445 PMC1181153

[B40] Sigel E Buhr A The benzodiazepine binding site of GABAA receptors Trends Pharmacol Sci 1997 18 425 429 9426470 10.1016/s0165-6147(97)01118-8

[B41] Rudolph U Möhler H GABA-based therapeutic approaches: GABAA receptor subtype functions Curr Opin Pharmacol 2006 6 18 23 10.1016/j.coph.2005.10.003 16376150

[B42] Marowsky A Fritschy JM Vogt KE Functional mapping of GABA A receptor subtypes in the amygdala Eur J Neurosci 2004 20 1281 1289 10.1111/j.1460-9568.2004.03574.x 15341600

[B43] Cohen AS Pfister BJ Schwarzbach E Grady MS Goforth PB Satin LS Injury-induced alterations in CNS electrophysiology Prog Brain Res 2007 161 143 169 full_text 17618975 10.1016/S0079-6123(06)61010-8

[B44] Mohler H GABA(A) receptor diversity and pharmacology Cell Tissue Res 2006 326 505 516 10.1007/s00441-006-0284-3 16937111

[B45] Obata K Oide M Tanaka H Excitatory and inhibitory actions of GABA and glycine on embryonic chick spinal neurons in culture Brain Res 1978 144 179 184 10.1016/0006-8993(78)90447-X 638760

[B46] Stein V Nicoll RA GABA generates excitement Neuron 2003 37 375 378 10.1016/S0896-6273(03)00056-4 12575946

[B47] Coulter DA Rafiq A Shumate M Gong QZ DeLorenzo RJ Lyeth BG Brain injury-induced enhanced limbic epileptogenesis: anatomical and physiological parallels to an animal model of temporal lob epilepsy Epilepsy Res 1996 26 81 91 10.1016/S0920-1211(96)00044-7 8985690

[B48] Golarai G Greenwood AC Feeney DM Connor JA Physiological and structural evidence for hippocampal involvement in persistent seizure susceptibility after traumatic brain injury J Neurosci 2001 21 8523 8537 11606641 10.1523/JNEUROSCI.21-21-08523.2001 PMC6762822

[B49] Hoskison MM Moore AN Hu B Orsi S Kobori N Dash PK Persistent working memory dysfunction following traumatic brain injury: evidence for a time-dependent mechanism Neuroscience 2009 159 483 491 10.1016/j.neuroscience.2008.12.050 19167462 PMC4264540

[B50] Thompson CL Razzini G Pollard S Stephenson FA Cyclic AMP-mediated regulation of GABA-A receptor subunit expression in mature rat cerebellar granule cells: Evidence for transcriptional and translational control J Neurochem 2000 74 920 931 10.1046/j.1471-4159.2000.0740920.x 10693922

[B51] Sperk G Schwarzer C Tsunashima K Kandlhofer S Expression of GABA(A) receptor subunits in the hippocampus of the rat after kainic acid-induced seizures Epilepsy Res 1998 32 129 139 10.1016/S0920-1211(98)00046-1 9761315

[B52] Huopaniemi L Keist R Randolph A Certa U Rudolph U Diazepam-induced adaptive plasticity revealed by alpha1 GABAA receptor-specific expression profiling J Neurochem 2004 88 1059 1067 10.1046/j.1471-4159.2003.02216.x 15009662

[B53] Vaknin UA Hann SR The α1 subunit of GABA_A _receptor is repressed by c-Myc and is pro-apoptotic J Cell Biochem 2006 97 1094 1103 10.1002/jcb.20708 16294320

[B54] Brooks-Kayal AR Shumate MD Jin H Rikhter TY Kelly ME Coulter DA Gamma-aminobutyric acid (A) receptor subunit expression predicts functional changes in hippocampal dentate granule cells during postnatal development J Neurochem 2001 77 1266 1278 10.1046/j.1471-4159.2001.00329.x 11389177

[B55] Poulter MO Brown LA Tynana S Willick G William R McIntyre DC Differential expression of alpha1, alpha2, alpha3, and alpha5 GABA-A receptor subunits in seizure-prone and seizure-resistant rat models of temporal lobe epilepsy J Neurosci 1999 19 4654 4661 10341263 10.1523/JNEUROSCI.19-11-04654.1999 PMC6782587

[B56] Rudolph U Crestani F Benke D Brunig I Benson JA Fritschy J-M Matertin JR Bluethmann H Mohler H Benzodiazepine actions mediated by specific γ-aminobutyric acid_A _receptor subtypes Nature 1999 401 796 800 10.1038/44579 10548105

[B57] McKernan RM Rosahl TW Reynolds DS Sur C Wafford KA Atack JR Farrar S Myers J Cook G Ferris P Garrett L Bristow L Marshall G Macaulay A Brown N Howell O Moore KW Carling RW Street LJ Castro JL Ragan CI Dawson GR Whiting PJ Sedative but not anxiolytic properties of benzodiazepines are mediated by the GABAA receptor α1 subtype Nat Neurosci 2000 3 587 592 10.1038/75761 10816315

[B58] O'Dell DM Hamm RJ Chronic post-injury administration of Suritozole, a negative modulator at the GABA receptor, attenuates cognitive impairment in rats following TBI J Neurosurg 1995 83 878 883 10.3171/jns.1995.83.5.0878 7472558

[B59] Folkerts MM Parks EA Dedman JR Kaetzel MA Lyeth BG Berman RF Phophorylation of calcium calmodulin-dependent protein kinase II following lateral fluid percussion brain injury in rats J Neurotrauma 2007 24 638 650 10.1089/neu.2006.0188 17439347

[B60] Kurz JE Parsons JT Rana A Gibson CJ Hamm RJ Churn SB A significant increase in both basal and maximal calcineurin activity following fluid percussion injury in the rat J Neurotrauma 2005 22 476 490 10.1089/neu.2005.22.476 15853464

[B61] Kim HS Choi HS Lee SY Oh S Changes of GABA-A receptor binding and subunit mRNA level in rat brain by infusion of subtoxic dose of MK-801 Brain Res 2000 880 28 37 10.1016/S0006-8993(00)02687-1 11032987

[B62] Berman RF Verweij BH Muizelaar JP Neurobehavioral protection by the neuronal calcium channel blocker Ziconotide in a model of traumatic diffuse brain injury in rats J Neurosurg 2000 93 821 828 10.3171/jns.2000.93.5.0821 11059664

[B63] Skeen GA White HS Twyman RE The dihydropine nitrendipine reduces N-methyl-D-aspartate evoked currents of rodent cortical neurons through a direct interaction with the NMDA receptor associated ion channel J Pharmacol Exp Ther 1994 271 30 38 7965728

[B64] Das P Bell-Horner CL Huang RQ Raut A Gonzales EB Chen ZL Covey DF Dillon GH Inhibition of type A GABA receptors by L-type calcium channel blockers Neuroscience 2004 124 195 206 10.1016/j.neuroscience.2003.12.005 14960351

[B65] Houlihan LM Slater EY Beadle DJ Lukas RJ Bermudez I Effects of diltiazem on human nicotinic acetylcholine and GABAA receptors Neuropharmacology 2000 39 2533 2542 10.1016/S0028-3908(00)00116-7 11044725

[B66] Wang L Greenfield LJ Jr Post-hypoxic changes in rat cortical neuron GABAA receptor function require L-type voltage-gated calcium channel activation Neuropharmacol 2009 56 198 207 10.1016/j.neuropharm.2008.07.004 PMC2628328 18674547

[B67] Lyons HR Land MB Gibbs TT Farb DH Distinct signal transduction pathways for GABA-induced GABA(A) receptor down-regulation and uncoupling in neuronal culture: A role for voltage-gated calcium channels J Neurochem 2001 78 1114 126 10.1046/j.1471-4159.2001.00501.x 11553685

[B68] Matlib MA Schwartz A Selective effects of diltiazem, a benzothiazepine calcium channel blocker, and diazepam, and other benzodiazepines on the Na+/Ca+ exchange carrier system of heart and brain mitochondria Life Sci 1983 32 2837 2842 10.1016/0024-3205(83)90319-3 6406780

[B69] Scanlon JM Brocard JB Stout AK Reynold IJ Pharmacological investigation of mitochondrial Ca(2+) transport in central neurons: Studies with CGP-3 an inhibitor of the mitochondrial Na(+)-Ca(2+) exchanger Cell Calcium 7157 28 317 327 10.1054/ceca.2000.0171 11115371

[B70] Sarnowska A Beresewicz M Zablocka B Domanska-Janik K Diazepam neuroprotection in excitotoxic and oxidative stress involves a mitochondrial mechanism additional to the GABAAR and hypothermic effects Neurochem Int 2009 55 164 173 10.1016/j.neuint.2009.01.024 19428822

[B71] Paquet-Durand F Gierse A Bicker G Diltiazem protects human NT-2 neurons against excitotoxic damage in a model of simulated ischemia Brain Res 2006 1124 45 54 10.1016/j.brainres.2006.09.077 17070504

[B72] Paquet-Durand F Bicker G Hypoxic/ischaemic cell damage in cultured human NT-2 neurons Brain Res 2004 1011 33 47 10.1016/j.brainres.2004.02.060 15140642

